# Rice cellulose synthase-like protein OsCSLD4 coordinates the trade-off between plant growth and defense

**DOI:** 10.3389/fpls.2022.980424

**Published:** 2022-09-26

**Authors:** Xiong Liu, Zhongliang Yin, Yubo Wang, Sai Cao, Wei Yao, Jinling Liu, Xuedan Lu, Feng Wang, Guilian Zhang, Yunhua Xiao, Wenbang Tang, Huabing Deng

**Affiliations:** ^1^ College of Agronomy, Hunan Agricultural University, Changsha, China; ^2^ Hunan Provincial Key Laboratory of Rice and Rapeseed Breeding for Disease Resistance, Changsha, China; ^3^ Hunan Hybrid Rice Research Center, Hunan Academy of Agricultural Sciences, Changsha, China; ^4^ State Key Laboratory of Hybrid Rice, Changsha, China

**Keywords:** rice, cellulose synthase-like D, trade-off, disease resistance, widely targeted metabolomics

## Abstract

Plant cell wall is a complex and changeable structure, which is very important for plant growth and development. It is clear that cell wall polysaccharide synthases have critical functions in rice growth and abiotic stress, yet their role in plant response to pathogen invasion is poorly understood. Here, we describe a *dwarf and narrowed leaf in Hejiang 19* (*dnl19*) mutant in rice, which shows multiple growth defects such as reduced plant height, enlarged lamina joint angle, curled leaf morphology, and a decrease in panicle length and seed setting. MutMap analysis, genetic complementation and gene knockout mutant show that *cellulose synthase-like D4* (*OsCSLD4*) is the causal gene for *DNL19*. Loss function of *OsCSLD4* leads to a constitutive activation of defense response in rice. After inoculation with rice blast and bacterial blight, *dnl19* displays an enhanced disease resistance. Widely targeted metabolomics analysis reveals that disruption of *OsCSLD4* in *dnl19* resulted in significant increase of L-valine, L-asparagine, L-histidine, L-alanine, gentisic acid, but significant decrease of L-aspartic acid, malic acid, 6-phosphogluconic acid, glucose 6-phosphate, galactose 1-phosphate, gluconic acid, D-aspartic acid. Collectively, our data reveals the importance of *OsCSLD4* in balancing the trade-off between rice growth and defense.

## Introduction

The growth of plants under natural conditions is constantly threatened by pathogens. Plant cell wall is a highly controlled and dynamic physiological structure, which is crucial to plant growth and development, and it is also the first front to fight against pathogens (Underwood, 2012; Bacete et al., 2018). Plant cell wall is mainly composed of cellulose, hemicellulose, pectin, lignin and glycoprotein (Somerville et al., 2004). As the most widely distributed and abundant polysaccharide, cellulose is produced by cellulose synthase (CESA) complexes (CSCs) on the plasma membrane (Lampugnani et al., 2019). The *CESA* superfamily also includes nine cellulose synthase-like (*CSL*) families, from *CSLA* to *CSLH*, and *CSLJ* (Yin et al., 2009). The *CSLA* family has been demonstrated to possess mannan and glucomannan synthase activity (Dhugga et al., 2004; Liepman et al., 2005; Goubet et al., 2009), while *CSLC* family members participate in the xyloglucan backbone synthesis (Cocuron et al., 2007; Kim et al., 2020). *CSLF* and *CSLH* exist only in grasses and are responsible for mixed linkage beta-glucan synthesis (Burton et al., 2006; Doblin et al., 2009; Jobling, 2015). *CSLD* gene family is the most similar *CSL* gene family to *CESA* family and also shows mannan synthase activity (Richmond and Somerville, 2000; Yin et al., 2011). In rice, a total of 45 *CESA* and *CSL* genes are identified, of which 11 were predicted to be *OsCESA* and 34 were predicted to be *OsCSL* (Wang et al., 2010). Except for a few genes, the role of most putative *OsCESA* and *OsCSL* genes in cell wall biosynthesis and remodelling is still elusive. *OsCESA4*, *OsCESA*7 and *OsCESA9* are not functionally redundant in deposition of cellulose in the secondary wall (Tanaka et al., 2003). *CslF6* mediates the biosynthesis of mixed linkage glucan in rice (Vega-Sánchez et al., 2012). *OsCSLD1*, ortholog of *Arabidopsis* gene *AtCSLD3*, is indispensable for root hair elongation and shows root-specific expression (Kim et al., 2007). *OsCSLD4* plays an essential role in plant architecture of rice by regulating cell wall polysaccharide synthesis, and also participates in plant response to salt stress (Li et al., 2009; Hu et al., 2010; Wu et al., 2010; Luan et al., 2011; Yoshikawa et al., 2013; Ding et al., 2015; Zhao et al., 2022).

Alterations in plant cell wall component have been proven to impose a significant influence on disease resistance. By screening mutants with changed disease resistance, many mutants are turn out to be impaired with genes involved in cell wall biosynthesis (Zhao and Dixon, 2014). For instance, *Arabidopsis* mutants *procuste1*/*isoxaben resistant 1*/*constitutive expression of VSP1* (*prc1*/*ixr1*/*cev1*), *irregular xylem1/cesa8* (*irx1*/*cesa8*), *irx3*/*cesa7* and *irx5*/*cesa4*, are defective in the biosynthesis of primary or secondary cell wall, in accompaniment with enhanced resistance to pathogens (Ellis et al., 2002; Hernández-Blanco et al., 2007). The rice loss-of-function mutant *cslf6* displays strengthened resistance to a virulent isolate of *Xanthomonas oryzae* pv. *oryzae* (*Xoo*; Vega-Sánchez et al., 2012).

In plants, enhanced defense is usually accompanied by reduced costs of growth and reproduction, which is called the growth-defense trade-off and is a subtle and adaptive mechanism under adverse conditions (Figueroa-Macías et al., 2021). There is no doubt that phytohormones play a crucial role in the potential conflict between immune and growth signals. The resistance phenotype of *prc1*/*ixr1*/*cev1* is related to the activation of ethylene (ET) and jasmonic acid (JA) signaling, while *irx1*/*irx3*/*irx5*-mediated resistance is partly attributed to constitutively activated abscisic acid (ABA) signaling pathway, not dependent on salicylic acid (SA), ET, and JA signaling (Ellis et al., 2002; Hernández-Blanco et al., 2007). Rice plants overexpressing *OsWRKY45* are strongly resistant to both *Magnaporthe oryzae* (*M. oryzae*) and *Xoo*, due to a synergistic interaction between the cytokinin and SA signalling pathways (Jiang et al., 2013; Akagi et al., 2014). *OsNPR1*-induced growth inhibition is caused by disrupting the auxin pathway through promoting IAA-amido synthase expression (Li et al., 2016). *OsBIHD1*, as a critical molecular switch, balances growth and immunity in rice by coordinating ethylene-brassinosteroid pathway (Liu et al., 2017). The application of trade-off genes in crop breeding is confronted with the challenge of improving both disease resistance and yield potential. However, some progress has been made in optimizing the trade-off between yield and resistance. Manipulation of *OsWRKY45* or *IPA1* expression by different promoters develops disease-resistant rice without growth penalty (Goto et al., 2015; Liu et al., 2019).

*OsCSLD4* is on the same allele as the reported gene *NRL1/ND1/SLE1/DNL1/OsCD1* (Li et al., 2009; Hu et al., 2010; Wu et al., 2010; Luan et al., 2011; Yoshikawa et al., 2013; Ding et al., 2015). Recently, *OsCSLD4* has been found to participates in rice salt stress response by mediating abscisic acid biosynthesis (Zhao et al., 2022). However, the function of *OsCSLD4* in rice response to biotic stress remains unclear. In this study, we described a dwarf and narrowed leaf mutant *dnl19* and found that *OsCSLD4* was the candidate for *DNL19*. Our observation indicated that loss of function of *OsCSLD4* compromised rice morphology, but greatly enhanced its resistance to pathogen. Therefore, *OsCSLD4* was suggested to be an important regulator of the growth–defense trade-off in rice.

## Materials and methods

### Plant materials and growth conditions

The *dnl19* mutant, initially identified from a T-DNA-inserted mutant pool, was in the *japonica* background of rice variety Hejiang 19 (*Oryza sativa* L. ssp. *japonica*). To construct *OsCSLD4* knockout transgenic plants, the rice variety Nipponbare (*Oryza sativa* L. ssp. *japonica*) was used for transformation. The *dnl19*, as the pollen acceptor, was crossed with wild type Hejiang 19. The first generation (F_1_) plants were self-pollinated, and the second generation (F_2_) was used for genetic analysis. All plants were grown in a phytotron at 24-32°C with 12 h light/12 h dark, or in an experimental field under natural open-air condition.

### Genetic transformation in rice

To complement the *dnl19* mutant, the coding sequences (CDS) of *OsCSLD4* was amplified and inserted into a binary vector pCAMBIA1300, under control of a maize ubiquitin promoter. The recombinant plasmid pCAMBIA1300-proUbi-OsCSLD4 was introduced into the *dnl19* mutant. The positive transformants were identified by screening resistance to Hygromycin B (31282-04-9, Roche).

The *OsCSLD4* knockout mutant was generated by Biorun Bio-tech. Co., Ltd. (Wuhan, China). The online tool DSDecodeM was used to analyze the sequencing results of *oscsld4* mutant lines (Liu et al., 2015b).

### Cross section and microscopy

Rice leaf blades and internodes were fully immersed in FAA fixative solution, dehydrated through a series of alcohol (30%, 50%, 75%, 85%, 90%, 95%, 100%) for one hour at each concentration, and then soaked in xylene for 3 times, 20 min each time, and finally embedded in paraffin. Tissues were sliced into 5 µm thick sections using a microtome (HistoCore AUTOCUT, Leica). Sections were put into the toluidine blue staining solution for about 2 minutes and observed under optical microscopy (Eclipse E100, Nikon).

### MutMap analysis

Rice leaf genomic DNA was extracted, according to the CTAB method. Four different DNA samples, including two parents and two bulked DNA pools, were used for whole-genome resequencing analysis. The DNA pool was composed of equal DNA of 25 F_2_ plants with dwarf or normal morphology. To obtain high quality data, raw data was filtered by Phred score (Qphred) to remove adapter and low-quality reads. Then clean data was compared to the Nipponbare reference genome (https://rapdb.dna.affrc.go.jp/) using BWA software (http://bio-bwa.sourceforge.net/). Duplicates of the blast result was removed by picard (https://broadinstitute.github.io/picard/). Variation in single nucleotide polymorphism (SNP) and insertion-deletion (InDel) was investigated using GATK HaplotypeCaller tool (https://gatk.broadinstitute.org/hc/en-us). SNP-index was calculated, taking 500 kb as window and 50 kb as step.

### RNA analysis

For transcriptome analysis, rice flag leaves of *dnl19* and WT at heading stage were used for total RNA extraction, with CTAB-PBIOZOL reagent. The quality and concentration of total RNA were checked by a Nano Drop (Thermo Fisher Scientific, USA). Three biological replicates per sample were sent to BGI Shenzhen Co., Ltd. (Shenzhen, China) for mRNA library construction and sequencing. The RNA-seq data was analyzed on the online Dr. Tom system from BGI (https://biosys.bgi.com/) and has been deposited in National Center for Biotechnology Information (NCBI) Sequence Read Archive (SRA) database with accession number PRJNA853163.

For defense-responsive gene expression analysis, rice leaves at seedling and tillering stages were collected and used for total RNA extraction. To investigate the influence of *Xoo* infection on *OsCSLD4* gene expression, leaves of Nipponbare at tillering stages were used for inoculation, and 2 cm leaf fragments next to cutting site were used for RNA extraction. RNA samples were reverse-transcribed with HiScript^®^ II Q RT SuperMix for qPCR (R223-01, Vazyme). The Hieff^®^ qPCR SYBR Green Master Mix (11201ES03, Yeasen) was used to perform real time-quantitative PCR (RT-qPCR) with the Real-Time System CFX96™ C1000 Thermal Cycler (BioRad), and gene expression was analysed by BioRad CFX Manager.

### Pathogen inoculation

The fourth leaf blades of rice seedlings at five leaf stage were used for inoculation by *M. oryzae* isolate 110-2 strain from Hunan. A punch inoculation method was used for rice blast inoculation (Liu et al., 2015a). The *M. oryzae* isolate was cultured on oatmeal agar for 15 days at 28°C before sporulation. Spores were collected and spore suspension was adjusted to 5×10^5^ spores/ml. The two injured dots on each leaf were dipped by 5 μl spore suspension. At 7 days post-inoculation (dpi), the disease lesion length was measured and DNA was extracted for analyzing the relative fungal biomass. Primers for the *pot2* gene of *M. oryzae* and the ubiquitin (*OsUbi*) gene of rice genome were previously described (Li et al., 2017).

*Xoo* PXO99 was cultured on PSA medium plates (10 g/L peptone, 10 g/L sucrose, 1 g/L glutamic acid and 15 g/L agar) at 28°C for 2 days before they were suspended in sterilized water (OD600 = 0.5). A leaf-clipping method was used to inoculate rice leaves as previously described (Xu et al., 2019). The disease symptom was documented at 2 weeks after inoculation.

### Metabonomic analysis

The aboveground tissues of rice were collected at tillering stage and sent to Biotree (https://www.biotree.cn) for metabolomic profiling. All samples were performed on an ultra-high-performance liquid chromatography (UHPLC) system with a Waters ACQUITY UPLC HSS T3 column coupled to a SCIEX QTRAP 6500+ mass spectrometer (AB Sciex, USA). The detailed experimental procedures and analytical methods were described (Zou et al., 2022).

## Results

### Pleiotropic phenotypes of rice *dnl19* mutant

**Figure 1:**
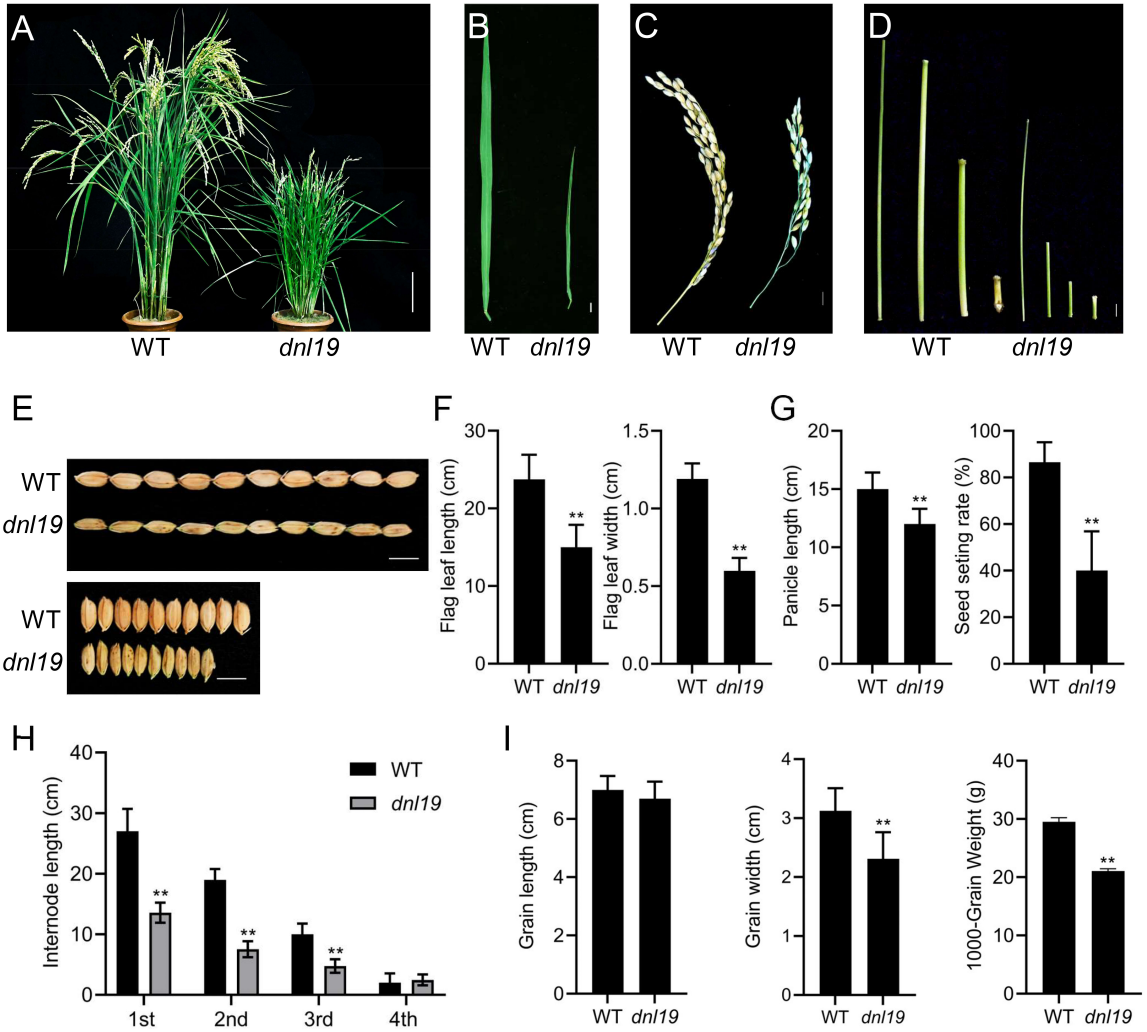
Characteristics of *dnl19* mutant. **(A)** Dwarfism was observed in *dnl19* plants at mature stage. Bar = 10 cm. **(B)** Flag leave of *dnl19* was slenderer than that of wild type (WT). Bar = 1 cm. **(C)** Panicle morphology was affected in *dnl19*. Bar = 1 cm. **(D)** The culm of *dnl19* was shorter than that of WT. Bar = 1 cm. **(E)** The grain shape was affected in *dnl19* plants. Bar = 1 cm. **(F)** Both the flag leaf length and width were reduced in *dnl19* mutant. Data are means ± SD (*n* ≥13, **P < 0.01, Student’s *t*-test). **(G)** The *dnl19* plants displayed a decrease in panicle length and seed setting rate. Data are means ± SD (*n*≥10, **P < 0.01, Student’s *t*-test). **(H)** The 1st, 2nd and 3rd internodes of *dnl19* were shortened. Data are means ± SD (*n*≥29, **P < 0.01, Student’s *t*-test). **(I)** Grain length, grain width and 1000-grain weight of WT and *dnl19* plants were statistically analyzed. Data are means ± SD (**P < 0.01, Student’s *t*-test).

We isolated a rice mutant (*dnl19*) displaying stunted growth from T-DNA insertion mutant seeds. During the whole developmental period, the *dnl19* mutant was shorter than the wild type (WT) (**Figure 1** **;** **Supplementary Figure 1**). At the seedling stage, *dnl19* plants were easily distinguished from WT because of their short stature, narrow leaves and enlarged lamina joint angle (**Supplementary Figures 1A–D**). At the heading and mature stages, the height of *dnl19* mutant was about half of that of the WT plants (**Figure 1A**; **Supplementary Figure 1E**). The flag leaf length and width, panicle length and internode elongation in *dnl19* mutant were also investigated. We found that flag leaf length was shortened in mutant, and leaf width was reduced to approximately 50% of normal leaf width (**Figures 1B, F** **)**. Panicle length and seed setting rate of *dnl19* plants were obviously decreased (**Figures 1C, G** **)**. The length of the first (1st), second (2nd), and third (3rd) internodes in mutant was decreased to 57%, 66%, and 80% of that of the WT plants, respectively (**Figures 1D, H** **)**. These results indicated that the dwarfism of *dnl19* was attributed to inhibited internode elongation, but not to fewer internodes. In addition, grain size was also affected in *dnl19* mutant. Compared with wild type plants, the *dnl19* plants had a significant decrease in grain width and 1000-grain weight (**Figures 1E, I** **)**.

### Altered leaf vein patterning and cell size of internode in *dnl19*

**Figure 2:**
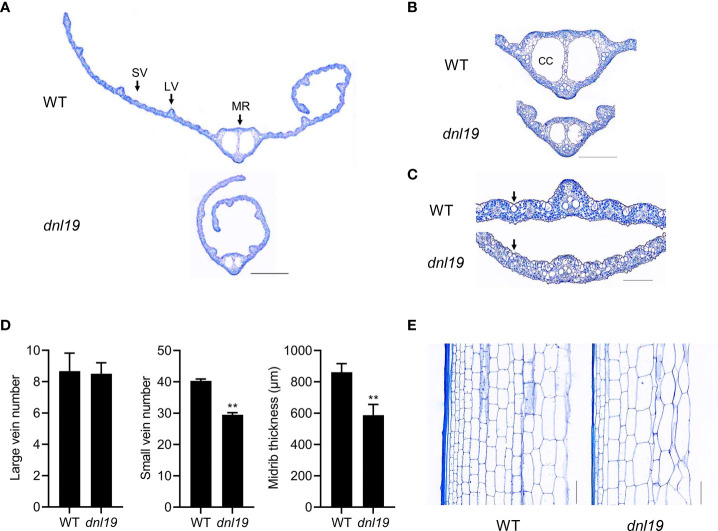
Effects of the *dnl19* mutation on leaf and culm structure. **(A)** Cross section of flag leaf blades in WT and *dnl19* plants. SV: small vein, LV: large vein, MR: midrib. Bar = 1 mm. **(B, C)** Magnified images of the leaf vein shown in **(A)**. CC: clear cell, Arrow: bulliform cell. Bar = 0.5 mm in **(B)** and 0.2 mm in **(C)**. **(D)** Number of large and small veins, and midrib thickness in leaf blades of WT and *dnl19* plants. Data are means ± SD (*n*=10, **P < 0.01, Student’s *t*-test). **(E)** Longitudinal section of the 2nd internodes at the mature stage from WT and *dnl19*. Bar = 0.1 mm.

To describe the *dnl19* mutant in detail, we analyzed the histology of leaf blade and internode sections in *dnl19* mutant. Leaf veins are important components of leaf morphology. We found that the number of large veins was not affected in mutant, while the number of small veins was decreased by 27%, resulting in the narrow-leaf phenotypes (**Figures 2A, D** **)**. Two large locules, called clear cells, are located in middle area of the midrib (Yamaguchi et al., 2004). The size of clear cells in *dnl19* was strikingly reduced, mostly contributing to a decrease in midrib thickness (**Figures 2B, D** **)**. Bulliform cells are mainly located on the upper epidermis of rice leaves and play a key role in regulating leaf rolling. We found that the *dnl19* mutant possessed significantly smaller bulliform cells in leaf vein areas than the wild type (**Figure 2C**), which could explain the easily-curled morphology of *dnl19* leaves. The examination of the longitudinal sections of the 2nd internodes revealed that the parenchymal cells were irregularly arranged and the number of horizontal cell layers were decreased in *dnl19*, and the cell size was enlarged compared with the wild type (**Figure 2E**). Therefore, these results suggested that the shortened length in the 2nd internode was due to a reduction in cell number, not in cell size.

### Isolation of genomic flanking sequences of T-DNA insertion in *dnl19* mutant

The *dnl19* mutant was obtained from the progeny of transgenic plants transformed with the pCAMBIA1300 binary vector. To identify the T-DNA insertion site of *dnl19* mutant, high-efficiency thermal asymmetric interlaced PCR (hiTAIL-PCR) procedure was applied (Liu and Chen, 2007). The result showed that the T-DNA element was inserted in the intron region of *CHR702* (LOC_Os06g08480) (**Supplementary Figure 2A**), whose protein product belonged to Chromodomain-Helicase/ATPase-DNA-binding domain (CHD) family. However, it is well documented that T-DNA insertion mutant and knockdown of *CHR702* did not cause any morphological differences (Hu et al., 2012). Besides, it was observed that the transgene-free *dnl19*, also had a dwarf morphology (**Supplementary Figure 2B**). Therefore, we concluded that *CHR702* was not the causal gene of *dnl19* mutant.

### Identification of the candidate region and the causal SNPs of *dnl19* mutant by MutMap analysis

When *dnl19* was crossed with the original strain (Hejiang 19), the F_1_ plants showed normal plant height relative to the WT (**Supplementary Figure 3A**). In F_2_ generation, the segregation model of normal stature and short stature was in good agreement with the expected single genetic ratio of 3:1, indicating that the *dnl19* phenotype was controlled by a single recessive gene (**Supplementary Table 1**). A MutMap approach was employed to identify the *DNL19* gene. Samples of Hejiang 19, *dnl19*, normal pool and dwarf pool were prepared for whole-genome resequencing analysis with 30×sequencing depth. The clean data was compared to the Nipponbare reference sequence and covered at least 97% of the rice genome. Taking 500 kb as window and 50 kb as step, SNP-index was calculated. According to SNP-index ≥0.6 of sliding window, the candidate interval was obtained on chromosome 9, 11 and 12 (**Supplementary Figure 3B**).

**Figure 3:**
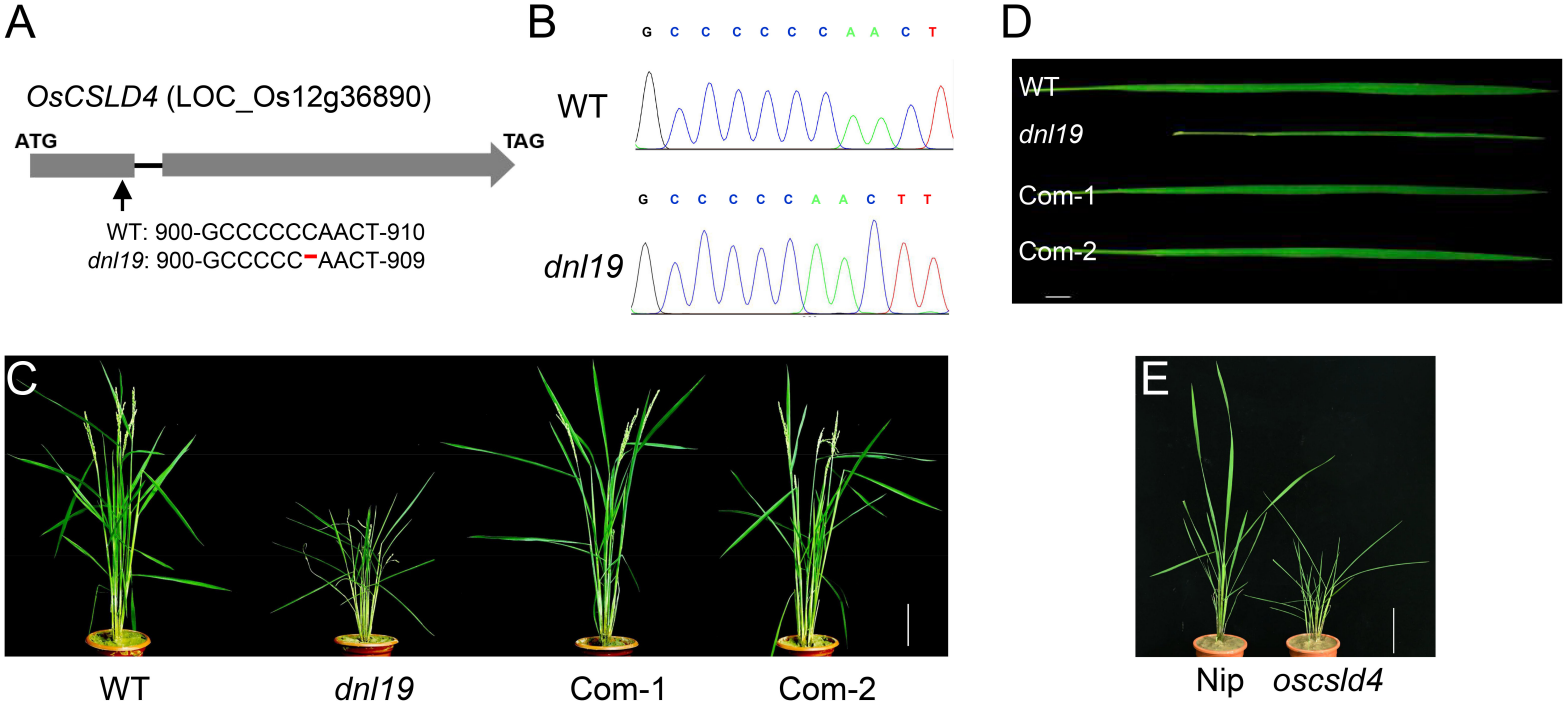
*OsCSLD4* is the causal gene of *dnl19* mutant. **(A)** Schematic diagram (not in scale) illustrates the InDel site in *OsCSLD4*. The arrow indicates the position of the *dnl19* mutation, and the red dash indicates a C base deletion. **(B)** Sequencing results of the *OsCSLD4* in wild type and *dnl19* mutant. **(C)** Leaf morphology of the wild plant, *dnl19* and functionally complemented plants (Com-1, Com-2). Bar = 1 cm. **(D)** Gross morphology of WT, *dnl19*, Com-1 and Com-2 at the maturing stage. Bar = 10 cm. **(E)** Gross morphology of Nipponbare (Nip) and *oscsld4* at the tillering stage. Bar = 10 cm.

There were 13 SNPs in the candidate region with ΔSNP-index (SNP-index difference of two pools) >0.6, most of which are located in the non-coding region (**Table 1**). We found that the SNP at nucleotide position 22,606,187 was in the first exon of the gene *OsCSLD4* (LOC_Os12g36890), previously reported to function in regulating cell wall polysaccharide synthesis (Luan et al., 2011). A C base deletion at codon 302, leading to a frame-shift and introducing a premature stop codon at nucleotide 985 of *OsCSLD4* CDS (**Figure 3A**). Sequencing analysis confirmed the mutation site and the C deletion could be the reasons for the abnormal phenotype of *dnl19* mutant (**Figure 3B**).

**Table 1:** Summary of candidate genes containing SNPs with ΔSNP-index>0.6 on genomic region of chromosomes.

Chromosome	Position	Annotation type	Annotation gene	ΔSNP-index
Chr9	11395079	3_prime_UTR_variant	LOC_Os09g18594	1
Chr9	11395082	3_prime_UTR_variant	LOC_Os09g18594	1
Chr9	11395085	3_prime_UTR_variant	LOC_Os09g18594	1
Chr9	11395529	3_prime_UTR_variant	LOC_Os09g18594	1
Chr11	5123314	downstream_gene_variant	LOC_Os11g09560	1
Chr11	9404100	upstream_gene_variant	LOC_Os11g16950	1
Chr11	25079488	upstream_gene_variant	LOC_Os11g41760	1
Chr12	1450543	upstream_gene_variant	LOC_Os12g03601	1
Chr12	6158352	upstream_gene_variant	LOC_Os12g11400	1
Chr12	6158415	upstream_gene_variant	LOC_Os12g11400	1
Chr12	22140068	upstream_gene_variant	LOC_Os12g36110	0.894736842
Chr12	22606187	frameshift_variant	LOC_Os12g36890	0.666666667
Chr12	22780656	downstream_gene_variant	LOC_Os12g37160	0.8

To rescue the various developmental defects of *dnl19* mutant, we expressed *OsCSLD4* in *dnl19* plants under the control of the maize ubiquitin promoter. After the gene was successfully introduced into the mutant *dnl19*, the phenotype of *dnl19* was completely inhibited. As for plant height and the morphological features of leaves, there is no difference between complementary plants and wild type plants (**Figures 3C, D** **)**. In addition, the CRISPR/Cas9 system was employed to generate mutant named *oscsld4* in the Nipponbare background. Similar to *dnl19*, the *oscsld4* in T_0_ generation also produced a dwarf plant with altered leaves (**Figure 3E** **;** **Supplementary Figure 3C**).

### Transcriptome analysis of *dnl19* mutant by RNA-seq

**Figure 4:**
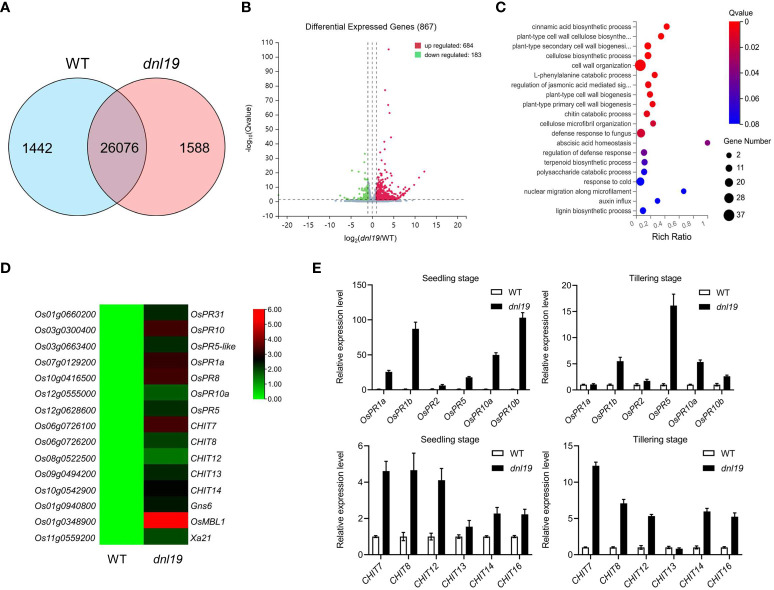
Global transcriptome analysis of *dnl19* mutant. **(A)** Venn diagram of common genes in *dnl19* and WT. **(B)** Volcano map of differentially expressed genes (DEGs) between *dnl19* and WT. **(C)** Significantly enriched GO terms of DEGs between *dnl19* and WT. GO terms belong to biological processes. **(D)** Heatmap of pathogen-associated genes transcription in *dnl19* and WT. **(E)** Investigation of the relative expression of pathogen-associated genes by qRT-PCR. The expression of *OsActin1* was used as an internal control.

In plants, it is common that impaired growth and development is accompanied by enhanced defense. To clarify whether the *OsCSLD4* was involved in rice defense response, transcriptome of *dnl19* and WT flag leaves at the heading stage were studied by RNA-seq analysis. 27,518 expressed genes were detected in WT and 27,664 expressed genes in *dnl19*, among which 26,076 genes were common ones (**Figure 4A**). Based on the threshold values of fold change ≥1.0 and *q*-value <0.05, a total of 867 genes were differentially expressed between *dnl19* and WT flag leaves, including 684 upregulated and 183 downregulated ones (**Figure 4B** **;** **Supplementary Table 3**). Besides cell wall organization, gene ontology (GO) classification of biological process of the DEGs were abundantly enriched in defense response (**Figure 4C**). And among those DEGs, we found that the transcripts of *pathogenesis-related* (*PR*) gene, *chitinase* gene, *Gns6* (Wang et al., 2021), *OsMBL1* (Han et al., 2019), and *Xa21* (Song et al., 1995), were highly upregulated in *dnl19* mutant (**Figure 4D**). The reported *narrow leaf and dwarf1* (*nd1*) was a *OsCSLD4* functional disruption mutant in Zhongxian 3037 indica variety (Li et al., 2009). Two-week old seedlings of *nd1* were treated with water (mock) or NaCl (150 mM NaCl) for two days, and sampled for RNA-seq analysis (Zhao et al., 2022). According to the available data, we found that lots of *PR* genes were identified in upregulated DEGs of *nd1* under mock treatment; *Gns6*, *OsMBL1*, and *Xa21* showed an increased transcript accumulation in *nd1* (**Supplementary Figure 4**). We checked the relative expression of some defense-responsive genes in *dnl19* and WT at both seedling stage and tillering stage using qPCR (**Figure 4E**). The *PR* genes, including *OsPR1a*, *OsPR1b*, *OsPR2*, *OsPR5*, *OsPR10a* and *OsPR10b*, were strikingly upregulated in 14-day-old plants of *dnl19*. And their expression, except for *OsPR1a* and *OsPR2*, was differently increased at tillering stage compared to control plants. We also found that transcripts of *CHITs*, other than *CHIT13*, were highly accumulated in *dnl19* at both seedling and tillering stage. Taken together, these results suggested that disruption of *OsCSLD4* in *dnl19* activated the expression of defense-responsive genes.

### Altered resistance to *M. oryzae* and *Xoo* in *dnl19* mutant

**Figure 5:**
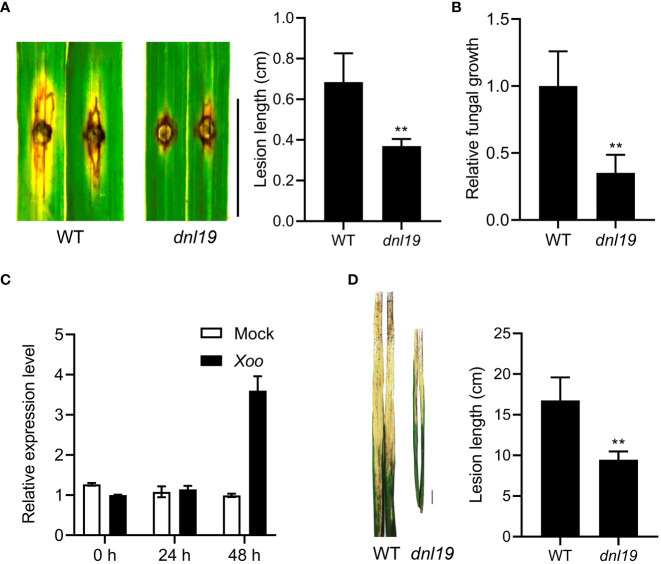
Phenotypical characterization of *dnl19* and wild type against *M. oryzae* and *Xoo*. **(A)** The *dnl19* mutant was inoculated with the *M. oryzae* isolate. Blast resistance was evaluated by punch inoculation. Lesion length were determined on leaves at 7 days after inoculation. Bar = 1 cm. Data are means ± SD (*n*=6, **P < 0.01, Student’s *t*-test). **(B)** Fungal growth was quantified by qPCR. Data are means ± SD (*n*=6, **P < 0.01, Student’s *t*-test). **(C)** The change of relative expression level of *OsCSLD4* in response to *Xoo* strain PXO99 treatment. The leaves of Nipponbare plants at tillering stage were used for inoculation. The expression of *OsActin1* was used as an internal control. **(D)** Phenotypes of disease reactions in *dnl19* flag leaves after inoculation with *Xoo* strain PXO99. Bar = 1 cm. Disease lesion length of wild type and mutant was measured at 2 weeks after inoculation. Data are means ± SD (*n*≥10, **P < 0.01, Student’s *t*-test).

Rice blast and bacterial blight are common and highly destructive diseases of rice. To investigate the ability against rice blast, *dnl19* was evaluated by punch inoculation at seedling stages. The results showed that the *dnl19* leaves had much less lesions after inoculation with *M. oryzae* isolate 110-2 strain (**Figure 5A**). The relative fungal biomass of each lesion was studied, and there were fewer fungi growing in the mutant leaves (**Figure 5B**).

In order to study the resistance of *dnl19* mutant to bacterial blight, we first tested whether *OsCSLD4* was responsive to *Xoo* strain PXO99 infection. We analyzed the expression of *OsCSLD4* in Nipponbare leaves inoculated with PXO99 at tillering stage. The result showed that the expression of *OsCSLD4* was markedly increase after 48 hours of treatment (**Figure 5C**). Then PXO99 was inoculated on the flag leaf of the *dnl19* mutant by tip-cutting method. At 2 weeks after inoculation, the *dnl19* showed significantly shorter lesion length than WT (**Figure 5D**). Taken together, these results suggested that loss of *OsCSLD4* gene function enhanced rice disease resistance.

### Metabolic profiling analysis of *dnl19* mutant

**Figure 6:**
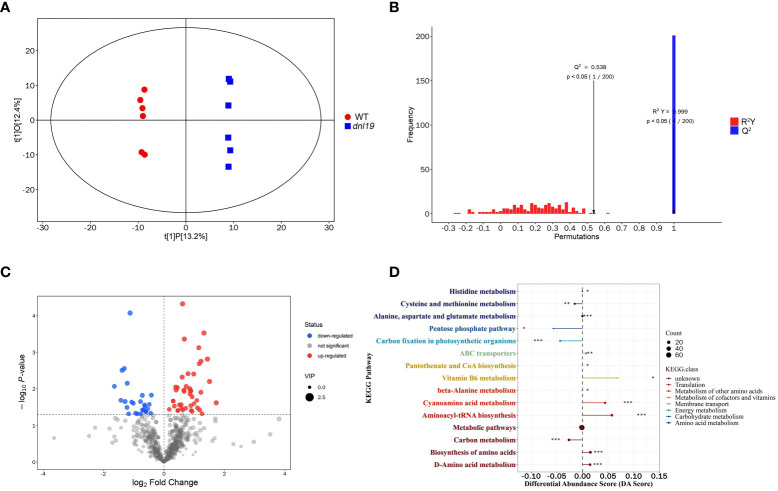
Widely targeted metabolomics analysis of *dnl19* mutant. **(A)** Scatter plot of OPLS-DA scores for WT group versus *dnl19* group. **(B)** Permutation tests of OPLS-DA model. The X-axis indicates the accuracy of the OPLS-DA model, and the Y-axis indicates the accuracy frequency of 200 models in 200 permutation tests. R^2^(Y) = 0.999, Q^2 =^ 0.538. **(C)** Volcano map of differential metabolites with VIP value ≥1 and *P*-value<0.05. The abscissa represents the fold change of each metabolite, and the ordinate represents the *P*-value by student’s *t*-test. **(D)** Analysis of overall changes of KEGG metabolic pathway. Each dot represents a metabolic pathway. The X-axis was the differential abundance (DA) score, and the Y-axis was the ID number of KEGG metabolic pathway. * represents significance. * *P* < 0.05, ** *P* < 0.01, *** *P* < 0.001.

To understand the metabolic effect of the *OsCSLD4* mutation, we applied widely targeted metabolomics using UHPLC-MS to investigate the chemical change of *dnl19* mutant. 678 metabolites were identified in total, including 118 flavonoids, 64 alkaloids, 64 terpenes, 58 phenols, 43 amino acids and derivatives, 27 fatty acyls, 24 steroids and steroid derivatives, 22 nucleotides and derivatives, 22 phenylpropanoids, 19 coumarins, and others (**Supplementary Table 4**). The *dnl19* group and the WT group were clearly separated by score plots of orthogonal partial least-squares discriminant analysis (OPLS-DA) (**Figure 6A**). The quality and validity of the OPLS-DA model were evaluated through 7-fold cross validation and permutation tests, respectively (**Figure 6B**). Any metabolite with variable importance in the projection (VIP) values ≥1, and *P*-value<0.05, was selected as differential metabolites. A total of 69 differential metabolites were identified; among them, there were 26 upregulated and 43 downregulated metabolites in *dnl19* mutant (**Figure 6C**; **Supplementary Figure 5A** **;** **Supplementary Table 5**). The upregulated metabolites included L-valine, L-asparagine, L-histidine, L-alanine, gentisic acid and other important metabolites. Meanwhile, downregulated metabolites included L-aspartic acid, malic acid, 6-phosphogluconic acid, glucose 6-phosphate, galactose 1-phosphate, gluconic acid, D-aspartic acid and so on. Based on the KEGG metabolic pathway analysis, the differential metabolites were enriched in 15 differentially metabolic pathways (**Supplementary Figure 5B**). Differential abundance score (DA Score) analysis was applied to reflect the overall change of all differential metabolites in a pathway. The pathways, including vitamin B6 metabolism, cyanoamino acid metabolism, aminoacyl-tRNA biosynthesis, biosynthesis of amino acids and D-amino acid metabolism, tend to be upregulated. And cysteine and methionine metabolism, pentose phosphate pathway, carbon fixation and carbon metabolism tend to be downregulated (**Figure 6D**).

## Discussion

Plants have evolved a precise mechanism to fine-tune the balance between growth and defense, which facilitates to ensure survival under adverse conditions. Cell wall biosynthesis has complex crosstalk with plant growth and defense. A significant body of genetic studies have shown that alteration of plant cell wall components affects the defense against pathogens (Zhao and Dixon, 2014). However, the detailed characterization of cell wall polysaccharide synthesis enzymes in rice disease response is still obscure. Previous studies have demonstrated that *OsCSLD4* not only plays important roles in rice growth and development, but also participates in rice salt stress response by mediating abscisic acid biosynthesis (Li et al., 2009; Hu et al., 2010; Wu et al., 2010; Luan et al., 2011; Yoshikawa et al., 2013; Ding et al., 2015; Zhao et al., 2022). Here, we describe in detail the characteristics of *dnl19* mutant, cloning of *DNL19* with MutMap approach, gene transcriptome analysis, pathogen inoculation and metabolomic analysis. All the data indicate that *OsCSLD4* is an important trade-off gene between rice growth and defense.

As we have illustrated, the *dnl19* mutant displayed a series of serious developmental defects (**Figure 1** **;** **Supplementary Figure 1**). Leaf morphology is one of the decisive factors of ideal plant architecture of rice. Moderate leaf curling is beneficial to photosynthesis and increase crop yield. The identified genes regulating leaf rolling are divided into five categories, among which, the changes of bulliform cell are considered to be the major cause (Xu et al., 2018). The leaf width of *dnl19* was significantly reduced, and its bulliform cells were smaller than those of wild type (**Figure 2**). Plant height is an important agronomic trait, which is closely related to lodging resistance. Most rice mutants with altered plant stature are identified to be associated with plant hormones gibberellins (GAs) and brassinosteroids (BRs) (Wang and Li, 2008). However, unlike GA deficient or BR deficient mutants, such as *sdg721*, *sdg705*, *d61* and *brd1-1* (Yamamuro et al., 2000; Hong et al., 2002; Jiang et al., 2018), there was no decrease in cell length in the internode region of *dnl19* mutant (**Figure 2**). It is speculated that the pathway related to *OsCSLD4* may be another way to control plant height, different from the plant hormone signal transduction pathway (Luan et al., 2011).

Many genes, which serve as the master regulators of the growth-defense trade-off, have been identified in rice. It was observed in transgenic rice overexpressing *OsWRKY45* and *IPA1* or knockout of *OsDOF11* and *OsALDH2B1*, that compromised plant morphology often brings about defense activation (Jiang et al., 2013; Akagi et al., 2014; Wu et al., 2018; Liu et al., 2019; Ke et al., 2020). We found that the expression levels of defense-related genes were markedly upregulated both in *OsCSLD4* knockout mutants *dnl19* and *nd1*, even if these two mutants were in distinct genetic background and samples for RNA-seq analysis were different (**Figure 4** **;** **Supplementary Figure 4**). In accordance with a constitutively activated defense response, the *dnl19* mutant had an enhanced resistance to rice blast and bacterial blight, two major biotic constraints that limit rice productivity (**Figure 5**).

The growth-defense trade-off process is considered to involve readjusting the allocation of resources to different pathways. Energy supply is essential for plants to combat with pathogens, which is achieved through primary metabolic activities (Bolton, 2009). Energy production involves many physiological processes, such as glycolysis pathway, pentose phosphate pathway, tricarboxylic acid (TCA) cycle and respiratory electron transport chain pathway (Less et al., 2011). Glucose 6-phosphate is formed in the first step of glycolysis, and 6-phosphogluconic acid is an intermediate product in pentose phosphate pathway, which begins with glucose 6-phosphate; malic acid, a four-carbon dicarboxylic acid, is a key intermediate of the TCA cycle (Fernie et al., 2004). We found that the relative contents of glucose 6-phosphate, 6-phosphogluconic acid, and malic acid were decreased in *dnl19* mutant (**Supplementary Figure 5** **;** **Supplementary Table 5**). It is possible that the energy saved by down regulating primary metabolism is transferred and used for defense response. Amino acids are closely related to plant defense, and the variation in amino acid levels is determined by pathogen species during plant-pathogen interaction (Ward et al., 2010). It has been reported that the level of asparagine was positively correlated with disease resistance in pepper and tomato (Hwang et al., 2011; Seifi et al., 2014). In *dnl19* mutant, L-asparagine was among the upregulated amino acids (**Supplementary Figure 5** **;** **Supplementary Table 5**). Secondary metabolites are small molecular compounds with biological activities, which regulate the growth and development of rice and promote disease and insect resistance (Wang et al., 2018). According to our results, the differential metabolites are mainly enriched in secondary metabolic pathways, including 17 flavonoids, 4 alkaloids, 6 terpenes, 6 phenols, and 2 steroids and steroid derivatives, suggesting that the dysfunction of *OsCSLD4* has a notable impact on the production of secondary metabolites.

*OsCSLD4* was related with hemicellulose polysaccharides, and mutation in *OsCSLD4* altered the content of xylose, cellulose, arabinose, homogalacturonan (Li et al., 2009). The *nd1* with changed cell wall composition reduced ABA content and impaired expression of ABA synthesis and response genes in an unknown mechanism (Zhao et al., 2022). It seems that *OsCSLD4* has opposite effects on the response of rice to abiotic and biological stresses, although the expression level of *OsCSLD4* is upregulated under salt stress or after pathogen inoculation (**Figure 5**). In *dnl19* mutant, transcripts of *pathogenesis-related* genes (*OsPR1a*, *OsPR1b*, *OsPR2*, *OsPR5*, *OsPR10a* and *OsPR10b*) and *chitinase* genes are highly accumulated, possibly supporting the notion that the basic energy of plants is more used for synthesis of defense responsive proteins. In addition, the enhanced disease resistance of *dnl19* mutant with a dwarf phenotype may also involve the activation of cell wall integrity (CWI) signaling. The plant CWI monitors the state of cell wall and triggers cell wall metabolism in response to adaptive changes in cell wall damage (Vaahtera et al., 2019). The *Arabidopsis* dwarf mutants, which are defective in lignin biosynthetic enzymes hydroxycinnamoyl CoA:shikimate/quinate hydroxycinnamoyl transferase (HCT) and cinnamoyl CoA reductase 1 (CCR1), exhibit extensive cell wall remodeling and release pectic oligosaccharide elicitors to activate defense-related genes expression (Gallego-Giraldo et al., 2020). In general, specific oligosaccharides are produced as a major plant-derived damage-associated molecular pattern (DAMP) during pathogen infection and can activate the plant immune response, such as 3^1^-β-D-Cellobiosyl-glucose and 3^1^-β-D-Cellotriosyl-glucose, which are released from the hemicellulose of rice cell wall during *M. oryzae* infection (Yang et al., 2021). However, it is unclear whether the *dnl19* or other *OsCSLD4* null mutants, defective in cell wall biosynthesis, will also release or provide precursors for oligosaccharide signal molecules to constitutively trigger plant defense response, which needs to be further investigated.

## Data availability statement

The datasets presented in this study can be found in online repositories. The names of the repository/repositories and accession number(s) can be found in the article/**Supplementary Material**.

## Author contributions

XL, ZY and WY performed experiments; XL and ZY analyzed data and wrote original draft; YW, SC, JL, XDL, FW, GZ, and YX provided suggestions and methodologies; YX, HD and WT designed experiments, reviewed and edited draft. All authors contributed to the article and approved the submitted version.

## Funding

This research was funded by the National Natural Science Foundation of China (32172078), the Science and Technology Innovation Program of Hunan Province (2021NK1001), Natural Science Foundation of Hunan Province (2021JJ40238 and 2022JJ30285), and Hunan Provincial Innovation Foundation for Postgraduate (CX20210665).

## Conflict of interest

The authors declare that the research was conducted in the absence of any commercial or financial relationships that could be construed as a potential conflict of interest.

## Publisher’s note

All claims expressed in this article are solely those of the authors and do not necessarily represent those of their affiliated organizations, or those of the publisher, the editors and the reviewers. Any product that may be evaluated in this article, or claim that may be made by its manufacturer, is not guaranteed or endorsed by the publisher.

## Supplementary material

Supplementary Table 4 All metabolites identified in WT and *dnl19* mutant.

Supplementary Table 5 Differential metabolites in WT and *dnl19* mutant.
