# Supplementary material for: Rice cellulose synthase-like protein OsCSLD4 coordinates the trade-off between plant growth and defense
Source: Front Plant Sci. 2022 Sep 26;13:980424. doi: 10.3389/fpls.2022.980424 (PMC9548992; doi:10.3389/fpls.2022.980424)
Supplement: Supplementary file 1 [file DataSheet_1.docx]

**Rice cellulose synthase-like protein OsCSLD4 coordinates the trade-off between plant growth and defense**

Xiong Liu^1,2†^, Zhongliang Yin^1,2†^, Yubo Wang^1,2^, Sai Cao^1,2^, Wei Yao^1^, Jinling Liu^1^, Xuedan Lu^1,2^, Feng Wang^1,2^, Guilian Zhang^1,2^, Yunhua Xiao^1,2^, Wenbang Tang^1,2,3,4^* and Huabing Deng^1,2^*

^1^College of Agronomy, Hunan Agricultural University, Changsha, China

^2^Hunan Provincial Key Laboratory of Rice and Rapeseed Breeding for Disease Resistance, Changsha, China

^3^Hunan Hybrid Rice Research Center, Hunan Academy of Agricultural Sciences, Changsha, China

^4^State Key Laboratory of Hybrid Rice, Changsha, China

**E-mail address for each author:**

Xiong Liu: xiongliu@whu.edu.cn; Zhongliang Yin: 1806890432@qq.com; Yubo Wang: wangyulive@163.com; Sai Cao: 1197530139@qq.com; Wei Yao: 411105639@qq.com; Jinling Liu: liujinling@hunau.edu.cn; Xuedan Lu: luxuedan1@126.com; Feng Wang: wangfenghifi@126.com; Guilian Zhang: zgl604@163.com; Yunhua Xiao: xyhkemy@163.com;

*Correspondence: Wenbang Tang: tangwenbang@163.com; Huabing Deng: denghuabing@126.com;

^†^ These authors have contributed equally to this work.

**
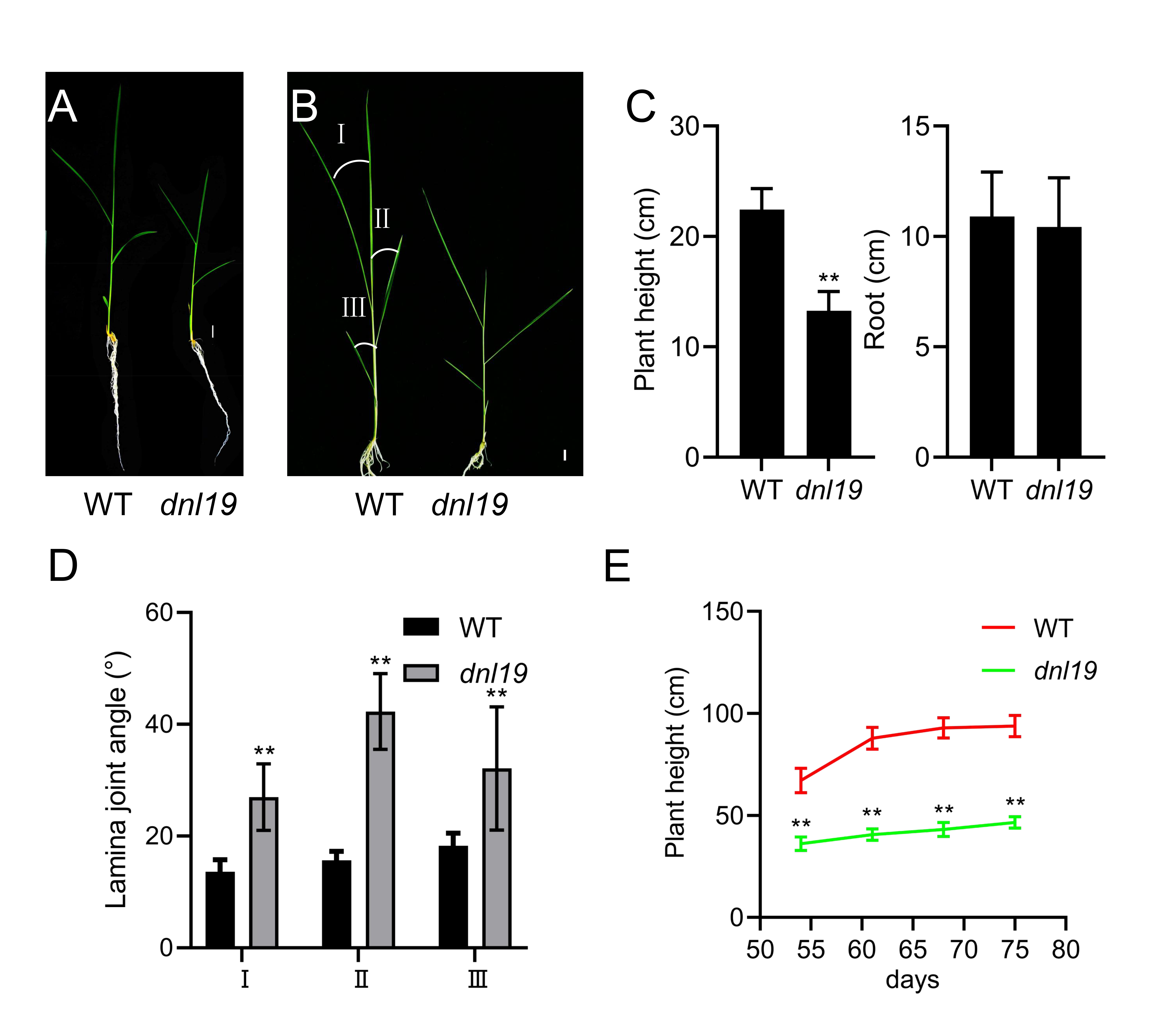
**

**Supplementary Figure 1.** Phenotypes of the *dnl19* mutant. (A) The *dnl19* mutant was shorter than WT at the 14th day after germination (DAG). Bar = 1 cm. (B) The *dnl19* mutant produced leaves with larger leaf angle at the 21th DAG. Bar = 1 cm. (C) Plant height and root length of WT and *dnl19* plants at the 14th DAG were compared. Data are means ±SD (*n*≥9, ***P*<0.01, Student’s *t*-test). (D) Leaf angle was measured in WT and *dnl19* plants at the 21th DAG. Ⅰ, Ⅱ and Ⅲ indicated the 3rd, 2nd and 1st leaf angle, respectively. Data are means ±SD (*n*=7, ***P*<0.01, Student’s *t*-test). (E) Dynamic variation of *dnl19* plant height was tracked from tillering stage to heading stage. Data are means ±SD (*n*≥22, ***P*<0.01, Student’s *t*-test).

**
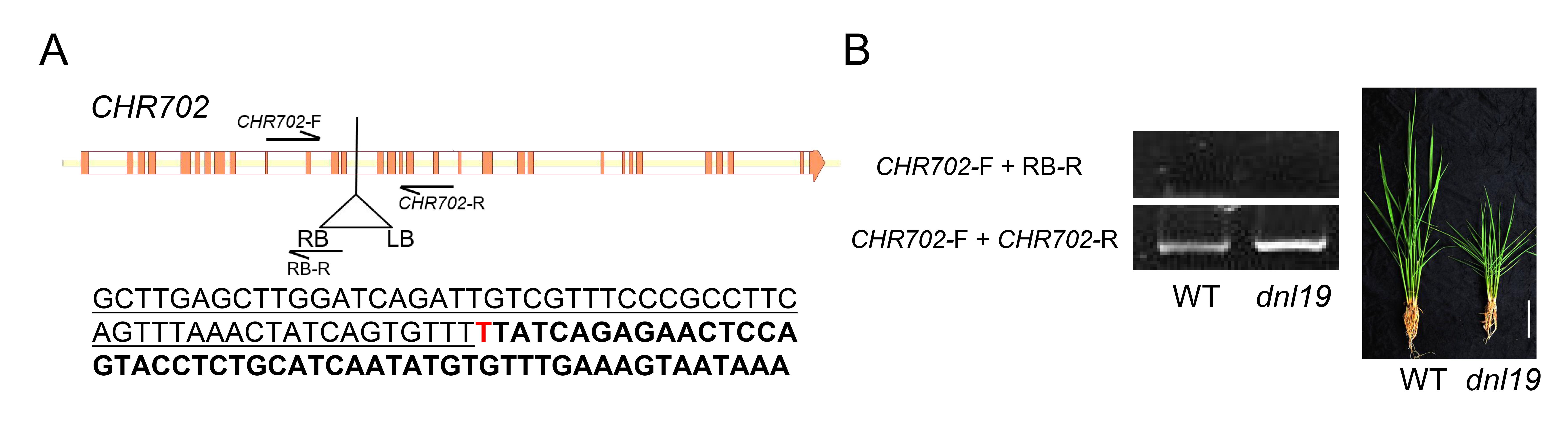
**

**Supplementary Figure 2.** The abnormality of *dnl19* was not caused by a T-DNA insertion event. (A) Schematic diagram (not in scale) illustrates the T-DNA insertion site in *CHR702*. The bases underlined indicate the T-DNA boundary sequence, the bold bases denote the genomic flanking sequence, and the red T indicates an additional base insertion. (B) Plant morphology of WT and *dnl19*. The *dnl19* was identified by a pair of primers. Bar = 10 cm.

**
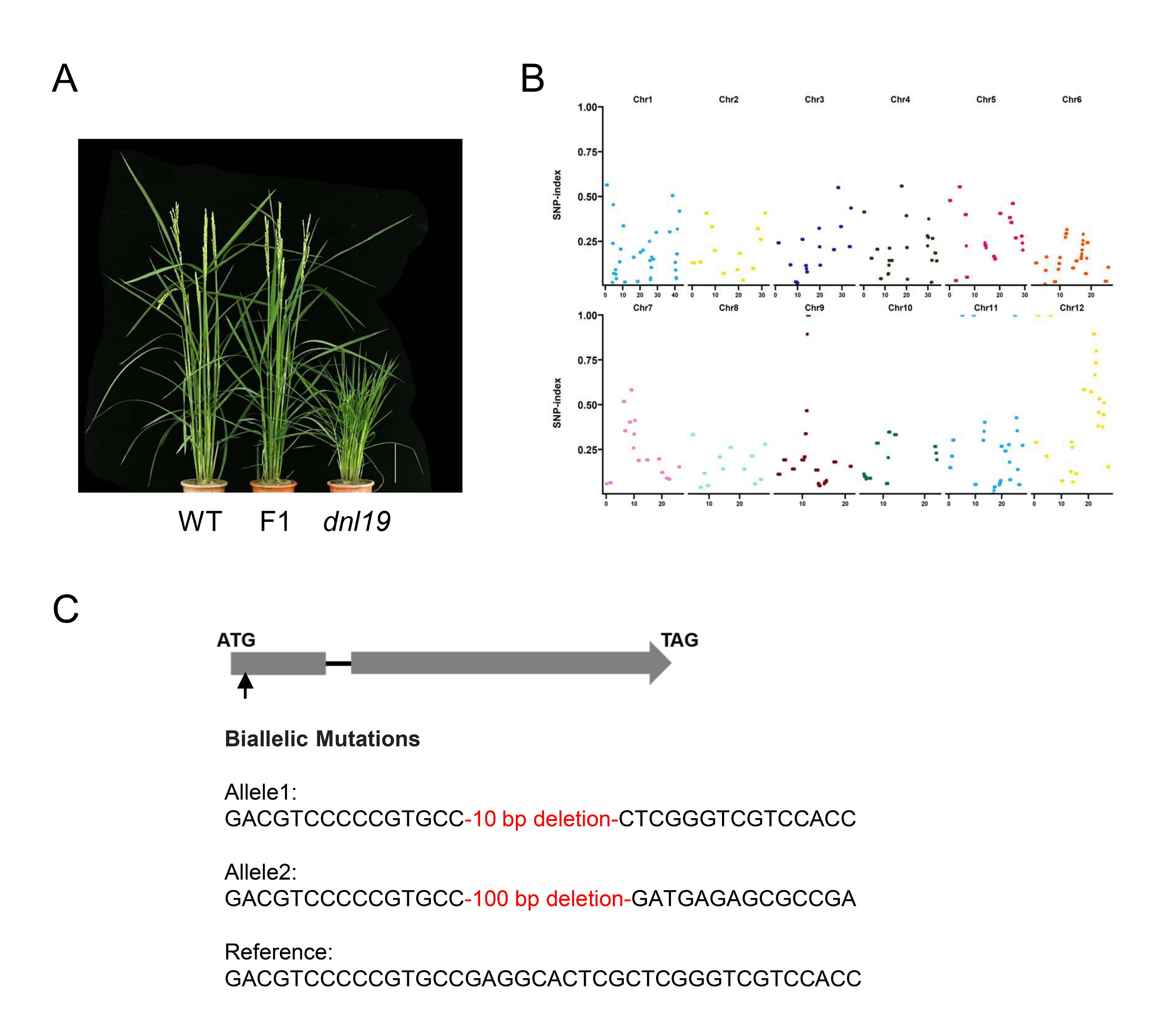
**

**Supplementary Figure 3.** Genetic and MutMap analysis of *dnl19*, and genomic edition of *OsCSLD4* knockout plant. (A) Gross morphology of Hejiang19 (WT), F_1_ plant between Hejiang19 and *dnl19*, and mutant *dnl19* at the booting stage*.* Bar = 10 cm. (B) Identification of candidate genomic region by MutMap approach. The X-axis represents the physical position of the chromosome, and the Y-axis represents the average value of SNP-index. (C) The targeted and edited DNA sequences in *OsCSLD4* gene were identified. The deletion is highlighted in red color.

**
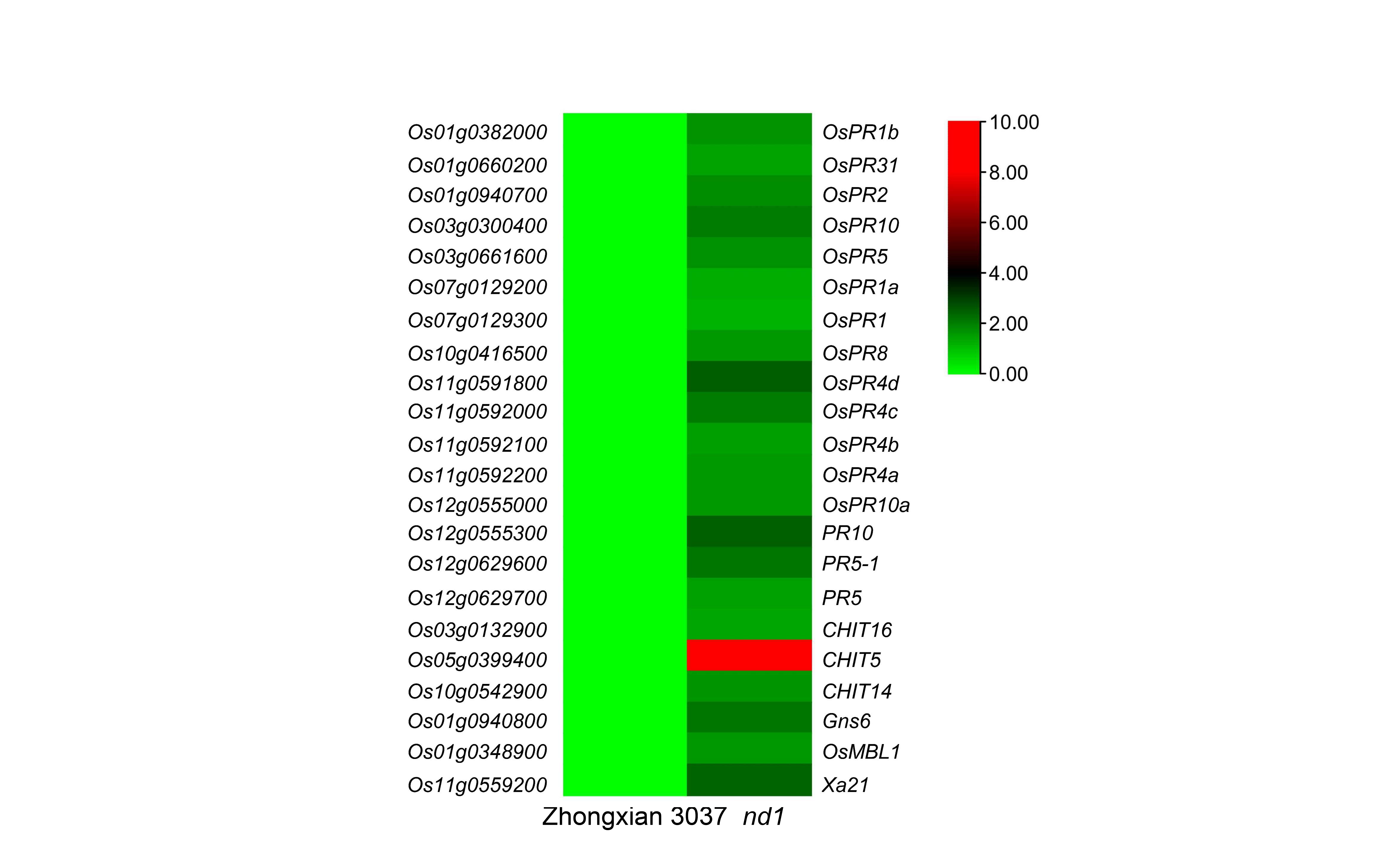
**

**Supplementary Figure 4.** Heatmap of pathogen-associated genes transcription in *nd1*.

**
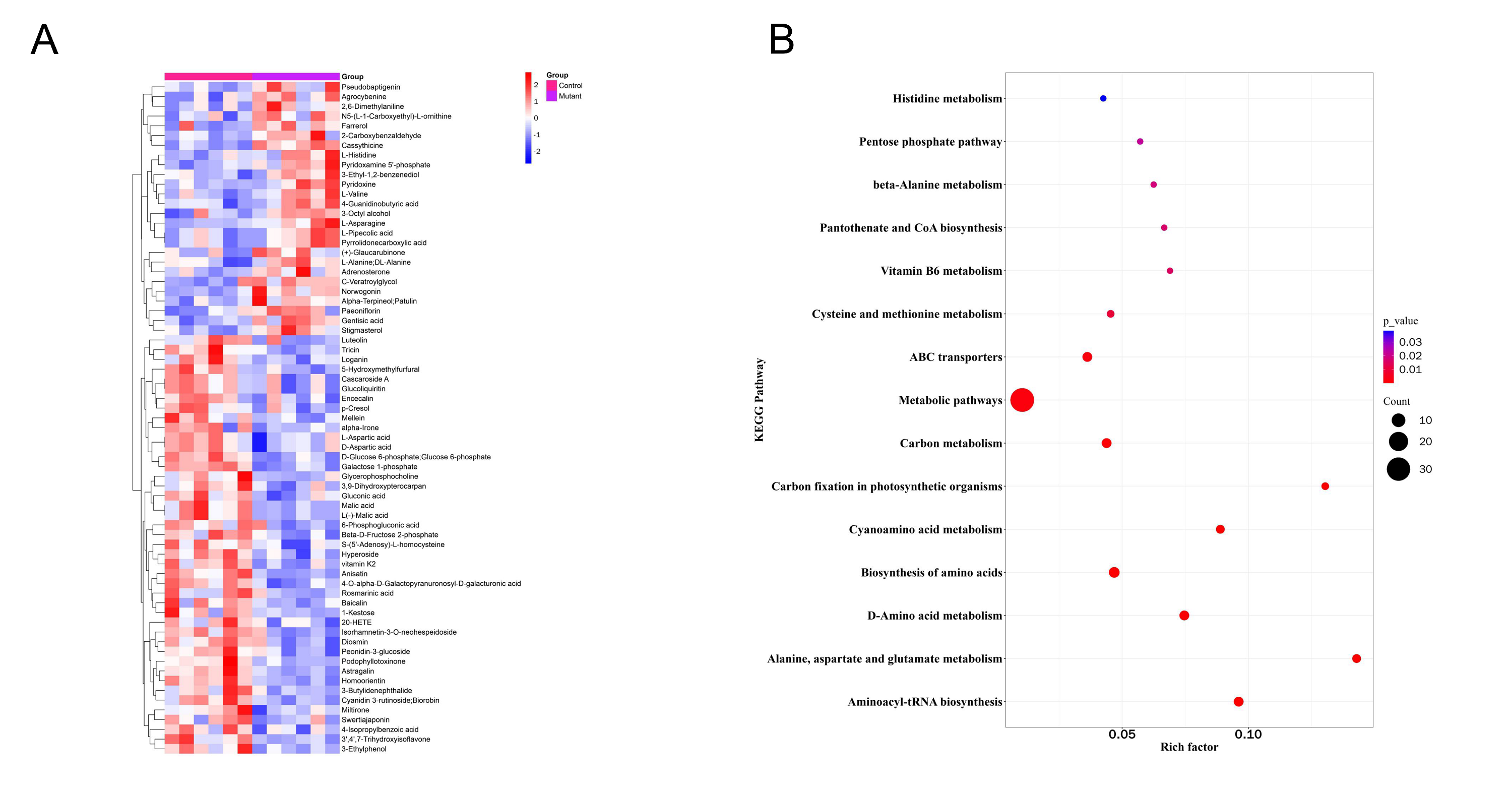
**

**Supplementary Figure 5.** Heat map and KEGG enrichment of differential metabolites for the *dnl19* and the wild type control. (A) Heat map showing metabolites with significant level changes (*p* < 0.05). The red color indicates significant increase of metabolites, the blue color, indicating significant decrease, and the white color indicates no significant difference. (B) Differential metabolites were enriched in 15 differentially metabolic pathways.

**Supplementary Table 1.** Segregation data for the *dnl19* mutant phenotype.

| Hybridized combination | No. of normal plants | No. of mutant plants | Expected Mendelian segregation ratio | *χ*^2^ | *P* value |
| --- | --- | --- | --- | --- | --- |
| *dnl19* (*♀*) × WT (*♂*) | 112 | 37 | 3: 1 | 2.588 | 0.108 |

**Supplementary Table 2.** Primers used in this study.

| Gene/Primer | Forward (5'-3') | Reverse (5'-3') |
| --- | --- | --- |
| **Homozygous (HM)/heterozygous (HZ) identification** | | |
| RB-R |  | TGCTAGAGCAGCTTGAGCTT |
| *CHR702* | AGATGAACCCGAAGAGCCTG | TTTCACCATCATCTTGTCAA |
| **qPCR** | | |
| *OsPR1a* | AAGCTGTACTGTCAGCCGTAT | GCATGTAACCACGAAGGACAG |
| *OsPR1b* | TATCCAAGCTGGCCATTGCT | GTTGTGGAGCCTCACGTAGT |
| *OsPR2* | CAAGATGGCAAAGCATGGCG | GCCGATAGATTGCACCACTGTA |
| *OsPR5* | TCGTCTTCTGCCCATAATGC | TGATTATCGATCAAGGTGTCGTT |
| *OsPR10a* | ATGAAGCTTAACCCTGCCGC | CCTCGAGCACATCCGACTTT |
| *OsPR10b* | TGAAGCTTAACCCTGCCGC | TTGCCCACCCTGCTCTTAAC |
| *CHIT7* | GTTCTACAAGCGCTACTGCG | ATCAACTAGGAAGGCGGGTAG |
| *CHIT8* | GACATGTTGGGCGTCAGCTA | TCCGATCAGTGGTTATCAGTGC |
| *CHIT12* | GCCAAGACCTCCTGTGGATA | GGCTTGGGCTCAATTCATGG |
| *CHIT13* | CCCCAATTCCACGATTAACGG | GGCATCACACACACAGTTGAC |
| *CHIT14* | CCAGTGGGGTTACTGCTTCA | TGGTAGTTCGATTGCCCTGT |
| *CHIT16* | TTCCCTAGATTCGGCACGAC | CAGTTGCCCATCCACCTGTA |
| *OsCSLD4* | CAGCCCCTACAGGTTGTTGA | ATGAGAAGGCGAACCACACC |
| *OsACTIN1* | GACCTTCAACACCCCTGCTA | GAGTCCAACACAATACCTGTGG |
| *Pot2* | ACGACCCGTCTTTACTTATTTGG | AAGTAGCGTTGGTTTTGTTGGAT |
| *OsUbi* | TTCTGGTCCTTCCACTTTCAG | ACGATTGATTTAACCAGTCCATGA |
